# Refractory Idiopathic Infantile Pyoderma Gangrenosum Successfully Treated With Infliximab: A Case Report

**DOI:** 10.1155/crpe/2259894

**Published:** 2026-10-22

**Authors:** Mio June Kurisawa, Tsunehisa Nagamori, Masayuki Sato, Emi Ishibazawa, Junki Tsuda, Mari Kishibe, Satoru Takahashi

**Affiliations:** ^1^ Department of Pediatrics Asahikawa Medical University Asahikawa Hokkaido Japan asahikawa-med.ac.jp; ^2^ Department of Dermatology Asahikawa Medical University Asahikawa Hokkaido Japan asahikawa-med.ac.jp

## Abstract

Pyoderma gangrenosum (PG) is a noninfectious neutrophilic dermatosis that can occur either idiopathically or secondary to various systemic conditions. PG is rare in childhood and even rarer in infancy. Due to its rarity, the pathophysiological mechanisms remain unclear. Although systemic glucocorticoids are considered the first‐line therapy, no standardized second‐line or subsequent therapeutic approach has been established, particularly for refractory cases. In this report, we present a case of infantile idiopathic PG showing sustained clinical improvement after the addition of the anti–tumor necrosis factor (TNF)‐alpha antibody infliximab (IFX). In addition, we conducted multiplex cytokine analysis to explore the patient’s inflammatory profile and provide preliminary observations regarding the inflammatory milieu associated with this condition. The patient, a 9‐month‐old infant, developed multiple pustules and ulcerative skin lesions on the face and extremities following Bacillus Calmette–Guérin (BCG) vaccination. Pathogen investigations were all negative, and the diagnosis of PG was made based on clinical findings, supported by histological features and exclusion of other conditions. After initial treatment with glucocorticoids and cyclosporine, remission was achieved; however, repeated relapses occurred. IFX was subsequently initiated, resulting in significant clinical improvement. Serum cytokine profiling at the onset revealed a broad elevation of proinflammatory cytokines, including soluble TNF receptors 1 and 2, with a pattern partially overlapping with that observed in Kawasaki disease. The clinical course and cytokine analysis results suggest that IFX may be a viable treatment option for refractory PG, even in infants.

## 1. Introduction

Pyoderma gangrenosum (PG) is an uncommon, noninfectious neutrophilic dermatosis characterized by recurrent, sterile, necrotic ulcers of unknown etiology. It can occur idiopathically or secondary to various systemic conditions, notably inflammatory bowel diseases, rheumatological disorders, and hematological malignancies [1]. PG is rare in childhood, accounting for only 4% of cases [2]. Due to its rarity, the precise disease mechanism is not well understood, and although systemic glucocorticoids are generally considered the first‐line therapy, there is limited clinical evidence regarding second‐line or subsequent treatment for refractory cases, especially in childhood. Here, we present a case of a 9‐month‐old boy diagnosed with idiopathic PG that was refractory to conventional interventions but showed successful improvement with the addition of an anti‐TNF‐alpha antibody. We also performed exploratory cytokine profiling of this patient to provide descriptive immunological context, together with a comparison with Kawasaki disease as a representative pediatric inflammatory condition.

## 2. Case Report

A previously healthy 9‐month‐old boy was referred to our hospital with multiple pustules and ulcers on his face and extremities. The patient had received the Bacillus Calmette–Guérin (BCG) vaccine at 7 months of age. Several days after vaccination, pustules developed at the inoculation site. These pustules rapidly expanded within a few days, progressing to ulceration, and subsequently disseminated to multiple areas within a month. He also developed a high fever. Due to a lack of response to empirical treatment with broad‐spectrum antibiotics and antituberculosis agents, he was admitted to our department.

At the time of admission, he was in good general condition but irritable due to pain. He presented with a 10‐cm diameter violaceous‐red flat‐elevated plaque with undermined ulcers surrounded by edema at the site of the BCG vaccination on his upper left arm (Figure 1A). He also had multiple ulcerative plaques and some impetigo‐like papules and pustules on his face and legs (Figure 1B,C). Notably, the sites of blood draw and venous access developed nodules within a few days, indicative of pathergy (Figure 1D).

**FIGURE 1:**
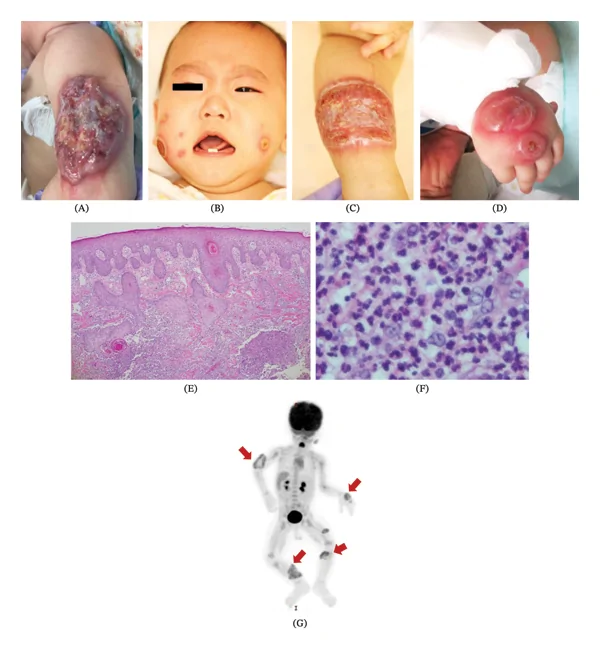
(A) Ulcerative lesion on the left upper arm corresponding to the BCG inoculation site. (B) Cutaneous manifestations of the face upon admission. (C) Ulcerative lesions on the lower right leg. (D) Blister‐like nodules with violaceous‐red edema present at the venous access site, suggestive of pathergy. (E) Histology of the ulcerative lesion of the lower leg (hematoxylin and eosin). (F) Pseudoepitheliomatous hyperplasia of the follicular epithelium and extensive neutrophil infiltration in the dermis were observed. (G) Whole‐body FDG‐PET imaging demonstrated FDG uptake corresponding to the cutaneous lesions, with no pathological uptake detected in other organs.

Blood tests revealed an inflammatory reaction with neutrophilic leukocytosis, but no abnormalities were found in lymphocytes or globulin subsets (Table 1). No pathogens were detected in cultures from the multiple lesions or by the PCR test for the *Mycobacterium tuberculosis* complex. A skin biopsy from an ulcer on the left lower leg showed dense inflammatory cell infiltrates throughout all layers of the dermis. The epidermis was markedly thickened, and folliculitis with pseudocarcinomatous proliferation of follicular epithelium within the dermis was also observed (Figure 1E). In high‐power fields, the infiltrating leukocytes were predominantly neutrophils (Figure 1F). No evidence of vasculitis was observed, and staining with Grocott, Ziehl–Neelsen, and auramine–rhodamine stains yielded negative results.

**TABLE 1:** Laboratory findings at admission.

WBC	35,670	/μL
Neutrophil	21,970	/μL
Lymphocyte	10,670	/μL
Monocyte	2250	/μL
Eosinophil	710	/μL
Hb	10.1	g/dL
Plt	101.6	× 10^4^/μL
PT‐INR	1.02	
APTT	28.2	sec
Fibrinogen	487	mg/dL
D‐dimer	2.0	mg/mL
AT‐III	117	%
CD3	46	% of lymphocyte
CD4	32	% of lymphocyte
CD8	16	% of lymphocyte
CD19	40	% of lymphocyte
Albumin	3.3	g/dL
AST	25	U/L
ALT	11	U/L
LDH	339	U/L
Creatinine	0.18	mg/dL
BUN	4.8	mg/dL
CRP	5.7	mg/dL
IgG	475.1	mg/dL
IgA	34.3	mg/dL
IgM	90	mg/dL
sIL‐2R	1094	U/mL
Ferritin	189.7	ng/mL
ANA	< 40	*x*
ESR	42	mm/h

*Note:* Hb: hemoglobin. Plt: platelet cells. PT‐INR: prothrombin time international normalized ratio. AT‐III: antithrombin III. AST: aspartate aminotransferase. ALT: alanine aminotransferase. LDH: lactate dehydrogenase. Ig: immunoglobulin. sIL‐2R: soluble interleukin‐2 receptor. ANA: antinuclear antibody.

Abbreviations: APTT, activated partial thromboplastin time; BUN, blood urea nitrogen; CD, cluster differentiation; CRP, C‐reactive protein; ESR, erythrocyte sedimentation rate; WBC, white blood cells.

No underlying condition, such as inflammatory bowel disease or malignancies, was identified through investigations including an FDG‐PET scan (Figure 1G) and a bone marrow examination. We also conducted whole‐exome sequencing to investigate potential genetic abnormalities associated with autoinflammatory syndromes known to be associated with PG, including PAPA, PASH, and PAPASH syndromes. Genes involved in inflammatory pathways, including inflammasome activation, IL‐1β signaling, and TNF‐related pathways, were also analyzed. However, no significant mutations that could explain the pathophysiology were detected. Thus, we diagnosed the patient with idiopathic PG. We started treatment with intravenous immunoglobulin (IVIG) and 2 mg/kg of prednisolone (PSL), considering the severe systemic inflammatory presentation and the need for rapid immunomodulatory intervention.

After initiation of the initial treatment, the emergence of new lesions and pathergy decreased; however, tapering of PSL led to a rebound increase in inflammatory markers. Thus, methylprednisolone (mPSL) pulse therapy was administered, and cyclosporine was subsequently introduced. In addition, diaminodiphenyl sulfone and colchicine were added to the treatment due to the recurrence of skin lesions. Although the patient partially responded to these treatments, there was a tendency for relapses at 3 months after the start of treatment during PSL tapering. Therefore, we initiated infliximab (IFX) at a dose of 5 mg/kg at baseline, at Weeks 2 and 6, and every 8 weeks thereafter. Following IFX initiation, the disease was controlled and remained in remission (Figure 2A). PSL, DDS, and colchicine were discontinued at 12, 13, and 15 months after disease onset, respectively. Thereafter, treatment was continued with IFX and cyclosporine. No serious adverse events or infections were observed during IFX therapy, and no new lesions appeared. The ulcers subsequently became completely epithelialized (Figure 2B), and his development was age appropriate. Following clinical resolution, subsequent vaccinations were carried out without any adverse events. IFX was discontinued 3 years after disease onset due to gastrointestinal adverse effects (intolerable nausea), and the patient has remained in remission thereafter.

**FIGURE 2:**
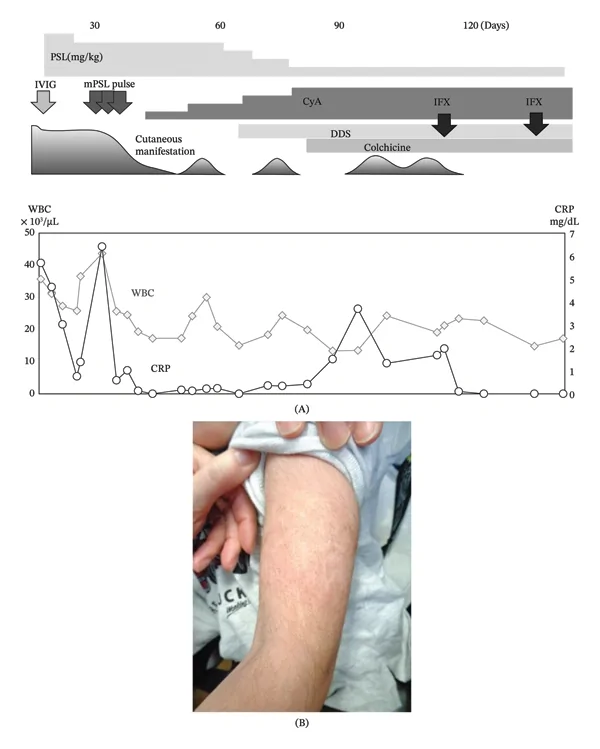
(A) Clinical course of the patient. The content of immunosuppressive interventions, extent of cutaneous manifestations, and time course of peripheral blood white blood cell count (left axis, × 10^3^/μL) and C‐reactive protein (right axis, mg/dL) are shown. (B) The skin lesion of the left upper arm healed with resultant scarring after IFX treatment initiation. Abbreviations: PSL, prednisolone; IVIG, intravenous immunoglobulin; mPSL, methylprednisolone; CyA, cyclosporin A; IFX, infliximab; DDS, diaminodiphenyl sulfone; WBC, white blood cells; CRP, C‐reactive protein.

### 2.1. Additional Examination

We also conducted cytokine profiling of this patient by evaluating serum cytokines using the Bio‐Plex multiplex assay system (Bio‐Rad, CA, USA). Human cytokine assay kits for interleukin‐6 (IL‐6) (#171B5006M), soluble tumor necrosis factor receptor (sTNFR)‐1 (#171BL034M), sTNFR‐2 (#171BL035M), interleukin‐18 (IL‐18) (#171B6023M), and interferon‐gamma–induced protein 10 (IP‐10) (#171B5020M) were individually purchased and evaluated simultaneously according to the manufacturer’s recommendations. The assays were performed on serum samples from this patient (two samples obtained before treatment initiation), healthy adults (*n* = 11), and patients with Kawasaki disease who had a typical clinical course (*n* = 15) as inflammatory disease controls. In this context, the term “typical clinical course” of Kawasaki disease refers to the following: (1) meeting more than 5 out of 6 of the clinical diagnostic criteria, (2) achieving improvement with the initial immunoglobulin therapy, and (3) the absence of residual coronary artery complications. As a result, we found the following: (A) no cytokines were elevated in healthy adults; (B) the cytokine profile in Kawasaki disease showed increases in a wide range of proinflammatory cytokines, including IL‐6, sTNFR‐1, sTNFR‐2, IL‐18, and the interferon‐responsive chemokine IP‐10; and (C) The cytokine pattern of this patient also showed a broad elevation of proinflammatory cytokines, which closely resemble that of typical Kawasaki disease (Figure 3).

**FIGURE 3:**
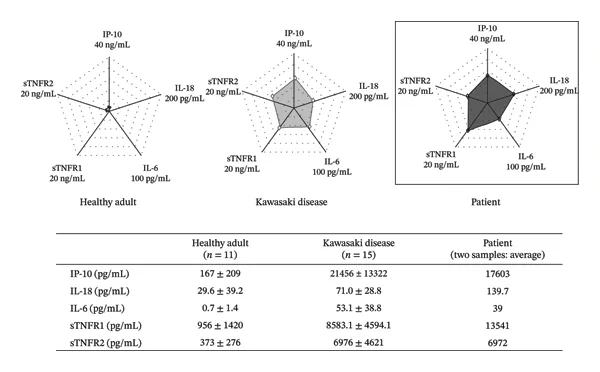
Cytokine profiling of this patient. Serum concentrations of interleukin‐6 (IL‐6), soluble tumor necrosis factor receptor (sTNFR)‐1, sTNFR‐2, interleukin‐18 (IL‐18), and interferon‐gamma–induced protein 10 (IP‐10) were evaluated in healthy adults (*n* = 11, left), patients with Kawasaki disease (*n* = 15, middle), and this patient (two samples taken before treatment initiation).

## 3. Discussion

We presented a case of an infant with idiopathic PG who showed sustained clinical improvement after the addition of IFX. Additionally, we provided an analysis of cytokine profiling, which may provide immunological context for the underlying inflammatory processes in infantile PG. PG in infancy is an exceptionally rare disease, with available information primarily limited to case reports and literature reviews [2]. Infantile PG has unique characteristics compared to adult cases: The proportion of comorbidities is lower (33% in infants, 75% in adults), while multiple or disseminated lesions and systemic signs such as fevers, anemia, and failure to thrive are more common [3]. Pathergy is also observed more frequently in infants (71% in infants, 33% in adults) [3]. Table 2 summarizes 17 cases of idiopathic infantile PG reported in the literature [3–16]. The age of onset ranges from 3 weeks to 11 months (median 8 months), with PG occurring during the neonatal period in two cases. Our case, presenting at 9 months with multiple ulcerative lesions, systemic signs, and pathergy, may represent the typical manifestations of infantile PG.

**TABLE 2:** Cases of idiopathic infantile pyoderma gangrenosum reported in the literature.

Reference number	Publication year	Age at onset	Sex	Glucocorticoids	Immunosuppressive agents	Biological agents
4	1982	9 months	Female	Prednisolone		
5	1994	3 weeks	Male	Prednisolone		
6	1996	7 months	Female	Prednisolone		
6	1996	9 months	Female	Prednisolone		
7	2000	10 months	Male	Betamethasone		
8	2006	8 months	Female	Prednisone	Tacrolimus (topical)	
9	2008	8 months	Male	Prednisolone	Cyclosporine A	
10	2009	4 months	Female	Prednisolone, methylprednisolone	Cyclosporine A	IFX
11	2012	4 months	Female	Prednisolone		
12	2013	23 days	Female	Prednisone		
13	2014	11 months	Male	Prednisolone	Cyclosporine A	IFX
14	2015	10 months	Female	Prednisolone, methylprednisolone	Cyclosporine A	ANK ⟶ IFX
15	2018	11 months	Female	Prednisolone	Tacrolimus (topical)	IFX
3	2022	8 months	Male	Methylprednisolone		ANK ⟶ IFX
16	2026	6 months	Male	Prednisolone, methylprednisolone		
Our case	2026	9 months	Male	Prednisolone, methylprednisolone	Cyclosporine A	IFX

*Note:* IFX: infliximab. ANK: anakinra.

The diagnosis of PG is primarily clinical, supported by histological findings and exclusion of alternative diagnoses, as no pathognomonic histological features have been established. Although the association between BCG vaccination and the onset of PG in our case could not be defined, there have been reports of infantile PG developing after minor trauma [9] and respiratory tract infections [4, 10]. It is possible that the vaccine acted as a trigger rather than as the primary cause of the disease. Regarding treatment, all patients received systemic glucocorticoids, including PSL or mPSL pulse therapy, with approximately half of the cases achieving remission with steroid monotherapy, while the other half were unresponsive or experienced relapses, necessitating combination therapy with immunosuppressants or biologic agents. For combination therapy, cyclosporine was the most used agent, administered in five cases. IFX was also used as a second‐line treatment in several cases. Notably, studies from recent years indicate an increasing preference for IFX in managing refractory pediatric PG cases [15, 17]. Interestingly, there have been two cases of infantile PG showing an incomplete response to anakinra, an anti–interleukin‐1 receptor antibody, with subsequent resolution after switching to IFX [3, 14]. This observation may suggest that IL‐1 inhibition alone is insufficient in some infantile PG cases and that TNF‐mediated inflammatory pathways may also play an important role.

As treatment options for adult PG, the efficacy of glucocorticoids and cyclosporine has been well demonstrated [18]. Also, TNF‐α inhibitors, particularly IFX, have become established as a first‐line option in specific clinical settings, supported by a randomized controlled trial [19, 20]. IVIG is positioned as an adjunct therapy in refractory PG [21]. However, in infantile PG, recent literature indicates that systemic corticosteroids remain the mainstay of first‐line therapy, while other agents such as cyclosporine, DDS, and biologics are typically reserved for steroid‐refractory cases [17].

Our treatment approach was consistent with this stepwise strategy for infantile PG. Although IVIG was introduced early in the clinical course because of its favorable safety profile in pediatric inflammatory diseases [22], treatment was initiated with glucocorticoids, and due to disease recurrence, additional therapies including cyclosporine, DDS, colchicine, and ultimately IFX were introduced. Thus, in the present case, the addition of IFX was associated with sustained clinical improvement, although the contribution of IFX cannot be distinguished from that of the concomitant immunosuppressive therapies. Nevertheless, the association between IFX initiation and subsequent disease control raises the possibility that TNF inhibition contributed to the favorable outcome in this refractory case.

We performed exploratory cytokine profiling in this case to characterize the inflammatory milieu and to provide retrospective immunological context for the clinical course. We found that a broad range of proinflammatory cytokines—including IL‐6, soluble TNF receptors, IL‐18, and IP‐10, an interferon‐inducible protein—was elevated. Although the precise pathology of PG remains unclear, it is hypothesized that excessive activation of the innate immune system may play a significant role based on histological findings and the nature of the comorbidities [1]. The cytokine profile observed in this patient was generally consistent with this hypothesis and showed similarities to a previously reported case of infantile idiopathic PG [14].

This cytokine profile showed partial overlap with that observed in Kawasaki disease. Kawasaki disease is a systemic vasculitis syndrome in children, in which overactivation of the innate immune system and the resulting overproduction of proinflammatory cytokines have been well characterized [23]. Because IFX has been demonstrated to be effective as a second‐line or later therapy for refractory Kawasaki disease [24], the similarity of these cytokine patterns in infantile PG may provide further rationale for using IFX in this condition.

However, these findings should be interpreted cautiously because they are derived from a single patient. In addition, the broad elevation of cytokines may reflect nonspecific inflammatory responses rather than a shared disease mechanism. Further investigation is required to establish the treatment strategy for infantile PG. Nevertheless, we believe that the clinical course and additional cytokine analysis in this patient suggest that TNF‐mediated inflammation may represent a relevant therapeutic target in refractory infantile PG, supporting the consideration of TNF inhibition as part of combination immunosuppressive therapy in selected cases.

## 4. Conclusion

This case demonstrated a favorable outcome of idiopathic infantile PG treated with an anti–TNF‐α antibody in addition to glucocorticoids and cyclosporine. Cytokine analysis revealed increases in multiple proinflammatory cytokines, resembling the pattern observed in Kawasaki disease, suggesting overlapping inflammatory pathways in this rare condition. Infliximab may represent a potential therapeutic option for infantile PG, although further studies are needed.

## Funding

This study was supported by the Japan Society for the Promotion of Science (JSPS) KAKENHI (grant number 24K11037).

## Ethics Statement

Cytokine analysis was conducted with approval from the Ethics Committee of Asahikawa Medical University (Accession No. #23183) and in accordance with the Declaration of Helsinki. Written informed consent was obtained from the patient’s parents for the publication of this case report and cytokine analysis. For the parents of patients with Kawasaki disease, informed consent was obtained through an opt‐out consent procedure on our website.

## Conflicts of Interest

The authors declare no conflicts of interest.

## Supporting Information

Additional supporting information can be found online in the Supporting Information section.

## Supporting information


**Supporting Information** CARE_checklist_PG_20260915.

## Data Availability

The data that support the findings of this study are available from the corresponding author upon reasonable request.

## References

[1] Flora A. , Kozera E. , and Frew J. W. , Pyoderma Gangrenosum: A Systematic Review of the Molecular Characteristics of Disease, Experimental Dermatology. (2022) 31, no. 4, 498–515, 10.1111/exd.14534. 35114021

[2] Kechichian E. , Haber R. , Mourad N. , El Khoury R. , Jabbour S. , and Tomb R. , Pediatric Pyoderma Gangrenosum: A Systematic Review and Update, International Journal of Dermatology. (2017) 56, no. 5, 486–495, 10.1111/ijd.13584. 28233293

[3] Lesmeister L. M. , Peitz J. , von Kleist-Retzow J. C. et al., Infliximab is an Appropriate Second-Line Therapy in Infants With Steroid Refractory Pyoderma Gangrenosum, Journal of the European Academy of Dermatology and Venereology. (2022) 36, no. 5, e376–e378, 10.1111/jdv.17921. 35037304

[4] Dick D. C. , Mackie R. M. , Patrick W. J. , and Goel K. M. , Pyoderma Gangrenosum in Infancy, Acta Dermato-Venereologica. (1982) 62, no. 4, 348–350, 10.2340/0001555562348350. 6183874

[5] Graham J. A. , Hansen K. K. , Rabinowitz L. G. , and Esterly N. B. , Pyoderma Gangrenosum in Infants and Children, Pediatric Dermatology. (1994) 11, no. 1, 10–17, 10.1111/j.1525-1470.1994.tb00065.x. 8170841

[6] al-Rimawi H. S. , Abuekteish F. M. , Daoud A. S. , and Oboosi M. M. , Familial Pyoderma Gangrenosum Presenting in Infancy, European Journal of Pediatrics. (1996) 155, no. 9, 759–762, 10.1007/BF02002902. 8874107

[7] Ravi M. D. and Sanju N. , Pyoderma Gangrenosum, Indian Pediatrics. (2000) 37, no. 11. 11086316

[8] Torrelo A. , Colmenero I. , Serrano C. , Vilanova A. , Naranjo R. , and Zambrano A. , Pyoderma Gangrenosum in an Infant, Pediatric Dermatology. (2006) 23, no. 4, 338–341, 10.1111/j.1525-1470.2006.00258.x. 16918628

[9] McAleer M. A. , Powell F. C. , Devaney D. , and O′Donnell B. F. , Infantile Pyoderma Gangrenosum, Journal of the American Academy of Dermatology. (2008) 58, no. 2 Suppl, S23–S28, 10.1016/j.jaad.2007.08.016. 18191692

[10] Rajan N. , Das S. , Taylor A. , Abinun M. , Spencer D. , and Carmichael A. , Idiopathic Infantile Pyoderma Gangrenosum With Stridor Responsive to Infliximab, Pediatric Dermatology. (2009) 26, no. 1, 65–69, 10.1111/j.1525-1470.2008.00825.x. 19250410

[11] Sarma N. , Bandyopadhyay S. K. , Boler A. K. , and Barman M. , Progressive and Extensive Ulcerations in a Girl Since 4 Months of Age: The Difficulty in Diagnosis of Pyoderma Gangrenosum, Indian Journal of Dermatology. (2012) 57, no. 1, 48–49, 10.4103/0019-5154.92678. PMC3312658 22470210

[12] Carneiro F. R. , Santos M. A. , Sousa B. A. , Nascimento Cdo S. , Amin G. A. , and Moutinho A. T. , Pyoderma Gangrenosum in a Newborn—Case Report, Anais Brasileiros de Dermatologia. (2013) 88, no. 6 Suppl 1, 173–175, 10.1590/abd1806-4841.20132601. PMC3876005 24346911

[13] Campos-Munoz L. , Conde-Taboada A. , Aleo E. , Toledano E. , and Lopez-Bran E. , Refractory Pyoderma Gangrenosum Treated With Infliximab in an Infant, Clinical and Experimental Dermatology. (2014) 39, no. 3, 336–339, 10.1111/ced.12297. 24635074

[14] Etzler C. , Cannon S. , and Hyde P. , Refractory Pyoderma Gangrenosum in an Infant, Pediatric Dermatology. (2015) 32, no. 1, 156–157, 10.1111/pde.12318. 24894462

[15] Crouse L. , McShane D. , Morrell D. S. , and Wu E. Y. , Pyoderma Gangrenosum in an Infant: A Case Report and Review of the Literature, Pediatric Dermatology. (2018) 35, no. 5, e257–e261, 10.1111/pde.13471. 29656404

[16] Tracy A. , Morrissette K. , and Kroshinsky D. , Infantile Pyoderma Gangrenosum: A Rare and Challenging Case Presentation With an Updated Literature Review, Pediatric Dermatology. (2026) 43, no. 2, 300–303, 10.1111/pde.70096. 41263180

[17] Kawabe S. , Iwata N. , Nishida E. et al., Combination Immunosuppressant Therapy for Rapidly Progressive Pyoderma Gangrenosum in a Child, Modern Rheumatology Case Reports. (2021) 5, no. 1, 137–140, 10.1080/24725625.2020.1749359. 33016252

[18] Dissemond J. , Marzano A. V. , Hampton P. J. , and Ortega-Loayza A. G. , Pyoderma Gangrenosum: Treatment Options, Drugs. (2023) 83, no. 14, 1255–1267, 10.1007/s40265-023-01931-3. PMC10511384 37610614

[19] Brooklyn T. N. , Dunnill M. G. , Shetty A. et al., Infliximab for the Treatment of Pyoderma Gangrenosum: A Randomised, Double Blind, Placebo Controlled Trial, Gut. (2006) 55, no. 4, 505–509, 10.1136/gut.2005.074815. PMC1856164 16188920

[20] Yamasaki K. , Yamanaka K. , Zhao Y. et al., Adalimumab in Japanese Patients With Active Ulcers of Pyoderma Gangrenosum: Final Analysis of a 52-Week Phase 3 Open-Label Study, The Journal of Dermatology. (2022) 49, no. 5, 479–487, 10.1111/1346-8138.16337. PMC9313851 35243682

[21] Ronicke M. , Sollfrank L. , Vitus M. V. et al., Intravenous Immunoglobulin Therapy for Pyoderma Gangrenosum: A Multicenter Retrospective Analysis in 81 Patients, American Journal of Clinical Dermatology. (2025) 26, no. 1, 139–146, 10.1007/s40257-024-00904-w. PMC11741994 39541069

[22] Danieli M. G. , Antonelli E. , Gammeri L. et al., Intravenous Immunoglobulin as a Therapy for Autoimmune Conditions, Autoimmunity Reviews. (2025) 24, no. 1, 10.1016/j.autrev.2024.103710. 39592027

[23] Hara T. Y. K. and Sakai Y. , The up-to-Date Pathophysiology of Kawasaki Disease, Clinical & Translational Immunology. (2021) . 10.1002/cti2.1284 PMC8109476 33981434

[24] Wang L. , He M. , Wang W. , Li S. , and Zhao G. , Efficacy and Safety of Infliximab in the Treatment of Kawasaki Disease: A Systematic Review and Meta-Analysis, European Journal of Pediatrics. (2024) 183, no. 4, 1765–1776, 10.1007/s00431-024-05437-2. 38240765

