# Supplementary material for: Refractory Idiopathic Infantile Pyoderma Gangrenosum Successfully Treated With Infliximab: A Case Report
Source: Case Rep Pediatr. 2026 Oct 22;2026:2259894. doi: 10.1155/crpe/2259894 (PMC13688240; doi:10.1155/crpe/2259894)
Supplement: Supplementary file 1 [file CRPE-2026-2259894-s001.pdf]

CARE
case report guidelines

CARE Checklist of information to include when writing a case report

(cc) BY-NC-ND

☑

Topic	Item	Checklist item description	Reported on Line
Title	1	The diagnosis or intervention of primary focus followed by the words "case report"	Page 1, lines 1-2
Key Words	2	2 to 5 key words that identify diagnoses or interventions in this case report, including "case report"	N/A
Abstract (no references)	3a	Introduction: What is unique about this case and what does it add to the scientific literature?	Page 2, lines 2-10
	3b	Main symptoms and/or important clinical findings	Page 2, lines 10-12
	3c	The main diagnoses, therapeutic interventions, and outcomes	Page 2, lines 12-16
	3d	Conclusion—What is the main "take-away" lesson(s) from this case?	Page 3, lines 1-2
Introduction	4	One or two paragraphs summarizing why this case is unique (**may include references**)	Page 4, lines 2-13
Patient Information	5a	De-identified patient specific information.	Page 5, line 2
	5b	Primary concerns and symptoms of the patient.	Page 5, lines 2-6
	5c	Medical, family, and psycho-social history including relevant genetic information	Page 5, line 2 ; Page 6, lines 8-13
	5d	Relevant past interventions with outcomes	Page 5, lines 6-8
Clinical Findings	6	Describe significant physical examination (PE) and important clinical findings.	Page 5, lines 9-14
Timeline	7	Historical and current information from this episode of care organized as a timeline	Figure 2A
Diagnostic Assessment	8a	Diagnostic testing (such as PE, laboratory testing, imaging, surveys).	Page 5, lines 15-18 ; Page 6, lines 1-12
	8b	Diagnostic challenges (such as access to testing, financial, or cultural)	N/A
	8c	Diagnosis (including other diagnoses considered)	Page 5, lines 15-18 ; Page 6, lines 1-13
	8d	Prognosis (such as staging in oncology) where applicable	N/A
Therapeutic Intervention	9a	Types of therapeutic intervention (such as pharmacologic, surgical, preventive, self-care)	Page 6, lines 13-18 ; Page 7, lines 1-7
	9b	Administration of therapeutic intervention (such as dosage, strength, duration)	Page 6, lines 13-15 ; Page 7, lines 4-13
	9c	Changes in therapeutic intervention (with rationale)	Page 6, lines 16-18 ; Page 7, lines 1-4
Follow-up and Outcomes	10a	Clinician and patient-assessed outcomes (if available)	Page 7, lines 5-13
	10b	Important follow-up diagnostic and other test results	N/A
	10c	Intervention adherence and tolerability (How was this assessed?)	Page 7, lines 8-12
	10d	Adverse and unanticipated events	Page 7, lines 8-12
Discussion	11a	A scientific discussion of the strengths AND limitations associated with this case report	Page 9, lines 2-4 ; Page 11, lines 5-9
	11b	Discussion of the relevant medical literature **with references**.	Page 9, line 5 - Page 12, line 5
	11c	The scientific rationale for any conclusions (including assessment of possible causes)	Page 12, lines 6-12
	11d	The primary "take-away" lessons of this case report (without references) in a one paragraph conclusion	Page 12, lines 9-12
Patient Perspective	12	The patient should share their perspective in one to two paragraphs on the treatment(s) they received	N/A
Informed Consent	13	Did the patient give informed consent? Please provide if requested	Yes ☑ No ☐
